# Sprifermin (rhFGF18) versus vehicle induces a biphasic process of extracellular matrix remodeling in human knee OA articular cartilage *ex vivo*

**DOI:** 10.1038/s41598-020-63216-z

**Published:** 2020-04-07

**Authors:** D. Reker, A. S. Siebuhr, C. S. Thudium, T. Gantzel, C. Ladel, M. Michaelis, A. Aspberg, M. W. Berchtold, M. A. Karsdal, A. Gigout, A. C. Bay-Jensen

**Affiliations:** 1 Biomarkers and Research Rheumatology, Nordic Bioscience A/S, Herlev Hovedgade 205–207, 2730 Herlev, Denmark; 2 Department of Biology, University of Copenhagen, Universitetsparken 13, 2100 Copenhagen Ø, Denmark; 3 Orthopedic Surgery Unit, Gentofte Hospital, Kildegårdsvej 28, 2900 Hellerup, Denmark; 4 Osteoarthritis Research, Merck KGaA, Frankfurter Strasse 250, 64293 Darmstadt, Germany; 5 Department of Clinical Sciences Lund, Lund University, Klinikgaten 28, 222 42 Lund, Sweden

**Keywords:** Prognostic markers, Drug regulation, Target validation

## Abstract

Sprifermin, recombinant human fibroblast growth factor 18 (rhFGF18), induces cartilage regeneration in knees of patients with osteoarthritis (OA). We hypothesized that a temporal multiphasic process of extracellular matrix (ECM) degradation and formation underlie this effect. We aimed to characterize the temporal ECM remodeling of human knee OA articular cartilage in response to sprifermin treatment. Articular cartilage explants from patients with knee OA (*n*_*patients*_ = 14) were cultured for 70 days, with permanent exposure to sprifermin (900, 450, 225 ng/mL), FGF18 (450 ng/mL), insulin-like growth factor-1 (100 ng/mL, positive control) or vehicle (*n*_*replicates/treatment/patient*_ = 2). Metabolic activity (AlamarBlue) and biomarkers of type IIB collagen (PIIBNP) formation (Pro-C2 enzyme-linked immunosorbent assay [ELISA]) and aggrecanase-mediated aggrecan neo-epitope NITEGE (AGNx1 ELISA) were quantified once a week. At end of culture (day 70), gene expression (quantitative reverse transcription polymerase chain reaction) and proteoglycan content (Safranin O/Fast green staining) were quantified. The cartilage had continuously increased metabolic activity, when treated with sprifermin/FGF18 compared to vehicle. During days 7–28 PIIBNP was decreased and NITEGE was increased, and during days 35–70 PIIBNP was increased. At end of culture, the cartilage had sustained proteoglycan content and relative expression of ACAN < COL2A1 < SOX9 < COL1A1, indicating that functional chondrocytes remained in the explants. Sprifermin induces a temporal biphasic cartilage remodeling in human knee OA articular cartilage explants, with early-phase increased aggrecanase activity and late-phase increased type II collagen formation.

## Introduction

Disease-modifying osteoarthritis drugs (DMOADs) that halt or prevent the progressive degeneration of joint tissues in osteoarthritis (OA), are urgently needed. Such drugs may either inhibit one or more of the catabolic processes of OA (anti-catabolic approach), induce regenerative processes in OA tissues (anabolic approach), or a combination of both. Several anabolic DMOADs have been investigated and shown anabolic effects on cartilage^1^, but it has proven challenging to provide convincing evidence of their clinically relevant effects on patients (reviewed by Hunter *et al*. 2011^2^).

Sprifermin is the first DMOAD candidate shown to induce cartilage regeneration in the knees of patients with OA. This was demonstrated in the 2-year primary endpoint data from the currently ongoing 5-year phase II clinical trial of sprifermin in knee OA^3^. Treatments with sprifermin consisting of one injection/week for 3 weeks resulted in a dose-dependent increase in total femorotibial joint cartilage thickness. Previous clinical trials and animal studies have also shown regenerative effects of sprifermin on joint cartilage^4–8^. Meanwhile, no local, systemic or serious safety concerns have been reported with intra-articular administration of sprifermin^3–5^.

The cartilage regenerative effect of sprifermin is driven by activation of the fibroblast growth factor receptor 3 (FGFR3) on the surface of chondrocytes^9,10^. Sprifermin is roughly five times more potent on this receptor compared to the natural ligand human fibroblast growth factor 18 (FGF18). The activation of FGFR3 induces proliferation, while maintaining the chondrogenic phenotype^10–13^. In addition, it induces formation of extracellular matrix (ECM) molecules type II collagen and aggrecan^8–12,14^.

We have previously shown a temporal ECM remodeling effect of sprifermin on bovine articular cartilage explants^12^. This study demonstrated that indeed chondrocytes could be reached and activated by sprifermin through the ECM of the explant and identified two response biomarkers: PRO-C2, reflecting type II collagen formation (the propeptide PIIBNP), and AGNx1, reflecting aggrecanase-mediated aggrecan degradation (the NITEGE fragment). Interestingly, NITEGE was released earlier than PIIBNP, indicating that early ECM degradation may be part of the regeneration process ultimately leading to increased ECM formation. Moreover, chondrocyte cultures (both monolayer and 3-dimensional) revealed an inverse linear correlation between proliferation and ECM formation, suggesting that chondrocytes cannot efficiently proliferate and produce ECM simultaneously^11^.

In summary, investigations on the FGF18 mode of action in chondrocyte monolayers and 3D cultures, as well as bovine explant show that FGF18 induce cell-proliferation and cartilage formation independently. Based on these observations, we investigated the effect of sprifermin on human OA cartilage and its ability to induce cartilage remodeling through a temporal multiphasic process of ECM degradation and ECM formation. ECM degradation and formation were monitored over time by measuring the release of NITEGE (aggrecanase-mediated aggrecan degradation) and PIIBNP (type IIB collagen formation), respectively. These biomarkers of ECM remodeling were identified in the previous bovine articular cartilage explants studies^12^. Ultimately, cartilage quality resulting from ten weeks of *ex vivo* culture was assessed by Safranin O/Fast green stainings (proteoglycan content) and gene expression analyses (COL2A1, ACAN, SOX9 and COL1A1 expression). Cartilage specimens from 14 patients were analyzed, to account for heterogeneity of the patient population.

## Methods

### Human knee OAarticular cartilage explants culture

To investigate the cellular and extracellular components of human knee OA articular cartilage in response to sprifermin, the well-described cartilage explant model was used^15–22^.The explants were cultured in serum-free medium to avoid interference from growth factors and proteolytical enzymes.

### Reagents

Human knee OA articular cartilage was obtained from the Department of Orthopedics at Gentofte University Hospital, Denmark, following international ethical guidelines for handling human samples and patient information (approved by the Danish National Committee on Biomedical Research Ethics, Department of Health, approval no. H D-2007-0084). All participants signed informed consent prior to enrolment in the study. The culture medium was composed of DMEM/Nutrient Mixture F-12 with GlutaMAX (DMEM/F12-GlutaMAX) with 1% Penicillin Streptomycin (PS) both obtained from Life Technologies. Phosphate buffered saline and human insulin-like growth factor-1 (IGF-1) were obtained from Sigma-Aldrich. FGF18 was obtained from Abcam. The sprifermin used was drug product formulated for intra-articular administration obtained from Merck KGaA together with the corresponding vehicle formulation. The vehicle formulation contained 7 mM Na2HPO4, 1 mM KH2PO4, 2.7 mM KCl (pH 7.3). Selection of doses was based on *in vivo* pharmacodynamic data published by Gigout *et al*. in 2017, where does between 100 and 1000 ng/mL were most effective^11^. The doses were confirmed and narrowed to 300 to 900 ng/mL by studies in bovine cartilage explant published by Reker *et al*. in 2017^12^.

### Tissue preparation

Human knee OA articular cartilage was harvested from macroscopically undamaged areas from femoral condyles of knee joint of OA patients undergoing knee replacement surgery due to knee OA. The cartilage was dissected from the bone by scraping a scalpel to the surface of the bone ensuring that all layers of the cartilage was included. Cartilage from the rim of the condyles were excluded. The scrap-offs were immediately transferred to sterile tubes containing culture medium and stored at 4 °C for maximum 2days.Age and gender of the patients is known to the surgeon however blinded to researchers, as depicted in the ethical permit issued by The Danish Science Ethical committee, Region of Copenhagen (H-D-2007-0084). Patient consent to have their remnant cartilage used for research without personal information attached.Cartilage plugs (i.e., explants) were isolated using a 3 mm diameter biopsy punch (Miltex), and the isolated explants (10–20 mg, 2–3 mm in height) were randomly distributed in 96-well culture plates with two explants per well. The explants were washed three times in culture medium and cultured in DMEM/F12-GlutaMAX with 1% PS at 37 °C, 5% CO_2_.

### Study design

Articular cartilage viable (based on metabolic activity) explants from 14 OA patients were included in the study.Explants from each patient were exposed to all treatments. From each patient, two plugs were added to each well, and two wells were used for each treatment arm (a total of 12 plugs per patients). However, due to limitations in tissue volume, a few exceptions had only one replicate well (see Supplementary Table 1, Additional File 3).The explants were cultured for 70 days. The 70-day incubation period was decided after several feasibility studies (data not shown). Once a week, conditioned medium was collected and stored at −20 °C for further analyses. Immediately after, freshly prepared medium with the following additions was added:Sprifermin (900, 450, 225 ng/mL), FGF18 (450 ng/mL), IGF-1 (100 ng/mL, positive control) orvehicle formulation (negative control). Hence, treatments were permanentlypresent in the medium. At termination (day 70), the explants were either fixed in 4% formaldehyde (*n*_*patients*_ = 8)or snap-frozen in liquid nitrogen (*n*_*patients*_ = 6) for further analyses.

### Metabolic activity

AlamarBlue reagent (Life Technologies) was used as a non-toxic reagent for quantifying the metabolic activity of the explants at baseline (day 0) and once a week during culturing in the conditioned medium. The assay was performed as previously described^12^.Only explants with measurable activity (n = 14) at baseline was continued to further experiments (Fig. S-6).

### Biochemical markers of ECM remodeling

#### PRO-C2enzyme-linked immunosorbent assay (ELISA)

The PRO-C2 ELISA (Nordic Bioscience) was used to quantify levels of type IIB collagen (PIIBNP) formation^17^. The PRO-C2 is a competitive ELISA that detects the PIIBNP propeptide (QDVRQPG), which is released during trimming of newly synthesized type II collagen by N propeptidases in the ECM. The assay was performed as previously described^12^.

#### AGNx1 ELISA

The AGNx1 ELISA (Nordic Bioscience) was used to quantify aggrecanase-mediated aggrecan degradation^22^. The AGNx1 is a competitive ELISA that detects the C-terminal peptide (NITEGE373) generated by aggrecanase cleavage. This ELISA recognizes all cleavage fragments that contain an exposed NITEGE epitope, thus including the 32 mer fragment (described by Lees *et al*.^23^), fragments with the G1 domain and even larger fragments with hyaluronic acid still linked to the G1 domain. The assay was performed as previously described^12^.

### Gene expression analyses

#### RNA extraction

The RNAqueous Kit (Ambion, AM1912) was used to extract total RNA from N_2_-preserved cartilage explants. Due to the low expected quantity of RNA in the cartilage explants, the four explants from each patient/treatment group were pooled. The RNAqueous Kit protocol guidelines for RNA extraction from “Frozen, hard-consistency, or RNase-rich tissue samples” were followed. Briefly, the frozen cartilage was pulverized in a pre-chilled bead mill, immediately transferred to the kit lysis/binding solution, and frozen at −80 °C. Prior to RNA extraction, samples were incubated at room temperature (RT) for 30 min and then centrifuged to remove debris. The standard kit protocol was followed for RNA extraction through glass fiber filters, and RNA yield and quality were assessed by NanoDrop spectrophotometry.

#### cDNA synthesis

First strand cDNA was synthesized using the Maxima First Strand cDNA Synthesis Kit for quantitative reverse transcription polymerase chain reaction (RT-qPCR) (Thermo Scientific, #K1641), following the supplied protocol. Due to low RNA yield from the cartilage explants, the maximum amount of RNA was added to the reaction.

#### RT-qPCR

TaqMan Gene Expression Assays were used together with TaqMan Fast Advanced Master Mix to quantify expression of the reference gene human EIF3I (assay ID Hs01116184_g1) and the target genes human COL2A1 (assay ID Hs00264051_m1), human ACAN (assay ID Hs00153936_m1),human SOX9 (assay ID Hs00165814_m1), and human COL1A1 (assay ID Hs00164004_m1).The supplied protocol was followed, again maximizing the amount of template cDNA, due to the low yield from cartilage explants. A non-template control was included for each assay, andan inter-plate control reaction quantifying EIF3I in a quality control sample was included on each plate. All reactions were run in triplicates. The RT-qPCR reactions were run on a StepOnePlus Real-Time PCR Instrument. The relative expression for each target gene in each sample were calculated by the 2^−ΔΔCt^ method, using EIF3I as reference gene. Levels were normalized to the vehicle group. Non-detectable reactions were excluded from analysis, and samples with non-detectable EIF3I were entirely excluded.

### Cartilage tissue staining

#### Safranin O/Fast Green

The histochemical staining Safranin O/Fast Green was used to visualize the proteoglycan content of formalin-fixed paraffin-embedded (FFPE) tissue slides of explants from 3 patients. Briefly, explants isolated at termination (day 70) were fixed in 4% formaldehyde (Merck Millipore) for 2 hours at RT and embedded in paraffin. Sections of 5 μm were cut across the cartilage layers, so that all slides included both the superficial, middle and deep layer of the cartilage. The sections were taken through de-paraffination and hematoxylin staining before staining first with Fast Green and then Safranin O. Sections were then dehydrated and mounted.

### Statistics

First, the dataset for each individual patient was calculated; each individual explant was baseline-corrected by dividing the value at each time point by the baseline value (day 0). Then, each individual explant was vehicle-corrected by dividing the baseline-corrected value at each time point by the corresponding baseline-corrected mean value of the vehicle-treated explants from the same patient. Second, the individual patient datasets were combined, by transferring the mean value for each patient and treatment group. The combined patient dataset was reported as median±95% confidence interval (*n* = 11).At single time points, medians of the treatment groups were compared to the vehicle group with Kruskal-Wallis test, using Dunn’s multiple comparisons test to correct for multiplicity. Statistical tests were performed in Graph-Pad Prism 6.0. Significance levels are indicated by exact *p* values.

## Results

### Model controls

Before further analyses were performed, two parameters were evaluated in all patients. First, the metabolic activity, assessed once a week, which in general was sustained at about 50% of the baseline (day 0) value after ten weeks of culture. Explants that dropped below 30% of their baseline value, were excluded from further analyses. Second, we evaluated PIIBNP response to IGF-1, which in most patient was increased to around 100 ng/mL (average of replicate explants) peaking within the first 3 weeks of culturing (day 7–21). In order to detect differences between treatment groups, only patients that had IGF-1 inducible levels of PIIBNP. In total, 3 patients were entirely excluded based on these two parameters (patient 3, 7 and 12). An overview of the number of explants and patients per treatment can be found in the additional files (Table S1).

### Sprifermin continuously increases metabolic activity of human OA cartilage explants

The metabolic activity was monitored in cartilage explants from 11 patients after 0, 7, 14, 21, 28, 35, 42, 49, 56, 63 and 70 days of culture with either sprifermin, FGF18 or vehicle treatment.Mean baseline levels of the metabolic activity patients were 10053 (3003) mAbs (Fig. S6).

**Figure 1:**
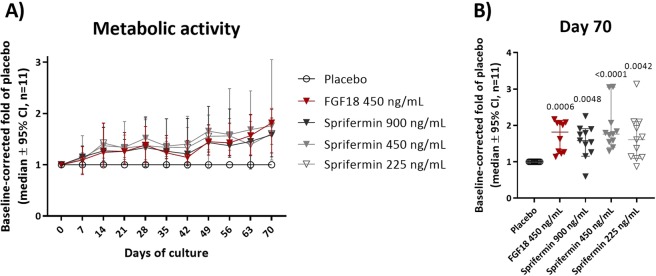
Metabolic activity (AlamarBlue) over time, patients combined (*n* = 11).Metabolic activity of human OA cartilage explants during ten weeks of culture. Patient 1, 2, 4, 5, 6, 8, 9, 10, 11, 13 and 14 were included. Explants were incubated 3 hours with 10% Alamar Blue® (**A**) The patient means (the mean of two replicate explants from each patient, calculated after baseline- and vehicle-correction) were combined and presented as median ±95% CI. (**B**) The medians ±95% CI of day 70 are presented. Significance level to vehicle was determined by Kruskal-Wallis test, using Dunn’s multiple comparisons test to correct for multiplicity, and *p*-values ≤ 0.05 are indicated above each treatment group.

Metabolic activity was continuously increased by sprifermin and by FGF18 compared to vehicle, when comparing the medians of the 11 OA patients (Fig. 1A). By termination of the study (day 70), all investigated doses of sprifermin and of FGF18 were significantly increased by 58% or more,compared to vehicle(*p* ≤ 0.005, Fig. 1B).When evaluating the individual patients, all patients responded with increased metabolic activity at all time points, but the exact pattern of the response (response-rate and time points of maximum effect) varied from patient to patient (Fig. S1). There were not significant differences observed between the different doses of sprifermin (Fig. 1B).

### Sprifermin induces biphasic ECM remodeling of human OA cartilage explants

To evaluate the temporal effect of sprifermin on ECM remodeling in human OA cartilage tissue, the biomarkers PRO-C2, AGNx1 and glycosaminoglycan (GAG; see Supplementary Methods, Additional File 1)were quantified in the conditioned media harvested from cartilage explants from 11 patients (GAG only in 5 patients) after 0, 7, 14, 21, 28, 35, 42, 49, 56, 63 and 70 days of culture with either sprifermin, FGF18 or vehicle treatment.

**Figure 2:**
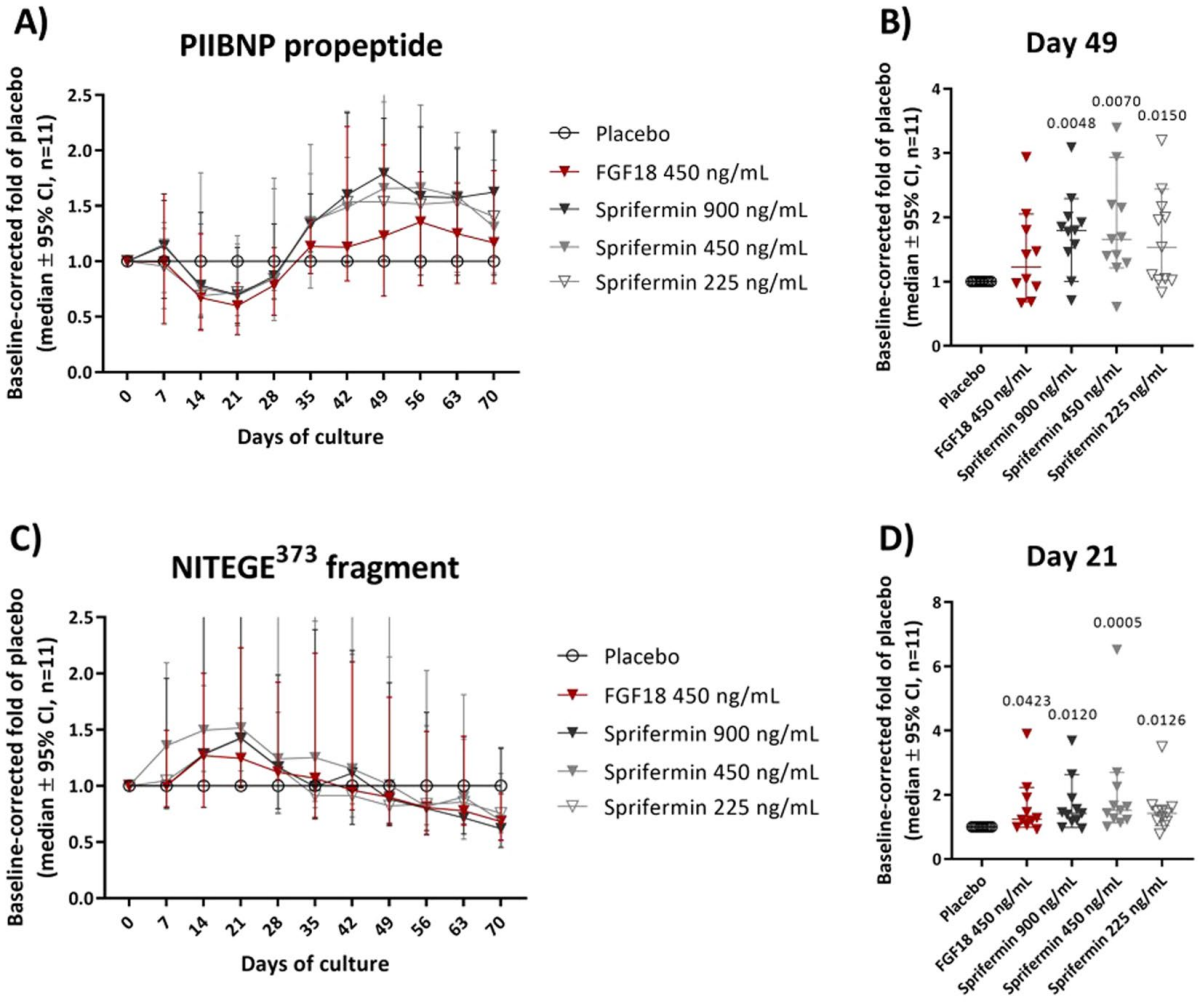
ECM remodeling (PIIBNP and NITEGE) over time, patient combined (*n* = 11).TypeII collagen formation (**A**,**B**) and aggrecanase-mediated aggrecan degradation (**C**,**D**) of human OA cartilage explants during 10 weeks of culture. Patient 1, 2, 4, 5, 6, 8, 9, 10, 11, 13 and 14 were included. The ECM remodeling was measured by PRO-C2 and AGNx1 ELISAs in conditioned media collected weekly during culturing. (**A**,**C**) The patient means (the mean of two replicate explants from each patient, calculated after baseline- and vehicle-correction) were combined and presented as median ±95% CI. (**B**,**D**) The medians ±95%CI of day 49 and 21 (peak response), for PIINAP and NITEGE releases respectively, are presented. Significance level to vehicle was determined by Kruskal-Wallis test, using Dunn’s multiple comparisons test to correct for multiplicity, and *p*-values ≤ 0.05 are indicated above each treatment group.

Type II collagen formation, reflected by PIIBNP, was modulated by sprifermin/FGF18 in a biphasic fashion compared to vehicle;PIIBNPwas decreasedduring early-phase culturing (day 7–28) and increased during late-phase culturing (day 35–70), when comparing the medians of the 11 OA patients to the vehicle control (Fig. 2A). The maximum early-phase decrease (day 21) was 31% for sprifermin (450 ng/mL, not significant) and 41% for FGF18 (*p* = 0.004). The maximum late-phase increase (day 49) was dose-dependent and significant for all investigated doses of sprifermin, compared to vehicle (*p* ≤ 0.015, Fig. 2B). The effect of FGF18 at day 49 (response peak) point was 23% increase (not significant), hence, much less pronounced than the comparative concentration of sprifermin (450 ng/mL), showing a 66% increase(*p* = 0.007, Fig. 2B). When evaluating the individual patients, all but one patient (patient 9) responded in a biphasic fashion (Fig. S2G). However, 3 patients (patient 9, 10 and 14) did not show the early-phase decrease (Fig. S2G,H,K), and 2 patients (patient 6 and 8) did not show the late-phase increase (Fig. S2E,F).

Aggrecanase-mediated aggrecan degradation, reflected by NITEGE, also revealed a biphasic response to sprifermin/FGF18 compared to vehicle. In contrast to PIIBNP, NITEGE was increased during early-phase culturing (day 7–28) and unchanged or decreased during late-phase culturing (day 35–70), when medians of the 11 OA patients were compared (Fig. 2C). The maximum early-phase increase (day 21) was significant for all investigated doses of sprifermin and FGF18, compared to vehicle (*p* ≤ 0.042).Although FGF18 had a significant effect of 21% (*p* = 0.042), it was clearly lower than the 52% increase by sprifermin 450 ng/mL (*p* = 0.0005) at day 21 (peak response) (Fig. 2D). When evaluating the individual patients, all but 2 patients (patient 2 and 8) responded in a biphasic fashion (Fig. S3F). Those 2 patients did not have the late-phase decrease/no change, but rather a continuous increase throughout culturing. In addition, one patient (patient 9) did not have the early-phase increase (Fig. S3G).

Glycosaminoglycan release, reflected by GAG, did not significantly respond to sprifermin compared to vehicleat any time point, when medians of 5 OA patients were compared (Fig. S4A). FGF18 induced a small, but significant increase in GAG during early phase (day 21; Fig. S4B).

### Cartilage was sustained throughout ten weeks of *ex vivo* culture

To evaluate the terminal (day 70) quality of the human OA cartilage tissue after ten weeks of *ex vivo* culture, gene expression analyses and histochemical staining were performed oncartilage explants harvested at study termination, after 70 days of culture with either sprifermin, FGF18 or vehicle treatment.

The relative expression of chondrocyte phenotype markers COL2A1 (encoding type II collagen), ACAN (encoding aggrecan) and SOX9 (encoding the chondrogenic transcription factor Sox9), and fibrocartilage marker COL1A1 (encoding type I collagen)was indicative of a hyaline cartilage. ACAN and COL2A1 were expressed at detectable levels in all patients, whereas SOX9 and COL1A1 were detected in most patients (Table 1). The relative expression of ACAN and COL1A1 was trending towards lower levels in the FGF18 and sprifermin group(Table 1). However, no significant difference in relative expression was observed betweensprifermin, FGF18, when comparing the medians of the 3–5 OA patients with detectable levels (Table 1).

**Table 1:** COL2A1, ACAN, SOX9 and COL1A1 fold expression (RT-qPCR) at day 70 (end of study).

	Vehicle	FGF18 450 ng/mL	Sprifermin 900 ng/mL	Sprifermin 450 ng/mL	Sprifermin 225 ng/mL	Kruskal-Wallis test (p-value)
COL2A1	1 *n* = 5/5	1.93(0.71–2.00) *n* = 3/3	0.81 (0.60–1.99) *n* = 5/5	0.81 (0.30–2.92) *n* = 5/5	1.41(0.39–3.65) *n* = 4/4	>0.1
ACAN	1 *n* = 5/5	0.84 (0.25–2.21) *n* = 3/3	0.57 (0.97–0.51) *n* = 5/5	0.34 (0.17–0.92) *n* = 5/5	0.63 (0.27–1.85) *n* = 4/4	0.041
SOX9	1 *n* = 5/5	1.01 (1.00–2.22) *n* = 3/3	1.22 (0.42–2.38) *n* = 5/5	0.65 (0.23–1.01) *n* = 4/5	2.04 (0.43–5.55) *n* = 4/4	>0.1
COL1A1	1 *n* = 4/5	0.40 (0.10–0.70) *n* = 3/3	1.91 (1.76–2.06) *n* = 3/5	0.66 (0.41–1.71) *n* = 3/5	0.25 (0.11–0.43) *n* = 4/4	0.012

Relative gene expression of human OA cartilage explants after ten weeks of culture. Patient 9, 10, 11, 13 and 14 were included. Gene expression was measured by RT-qPCR on RNA extracts of N2-preserved explants from termination of the experiment (day 70). All replicate explants for each patient and treatment were pooled for RNA extraction, resulting in 25 samples (5 patients x 5 treatments). The relative expression for each target gene (COL2A1, ACAN, SOX9 and COL1A1) in each sample was calculated by the 2^−ΔΔCt^ method, using EIF3I as reference gene. Values are median (range), and the no. of samples with a detectable expression level/no. of samples quantified by RT-qPCR is indicated.

**Figure 3:**
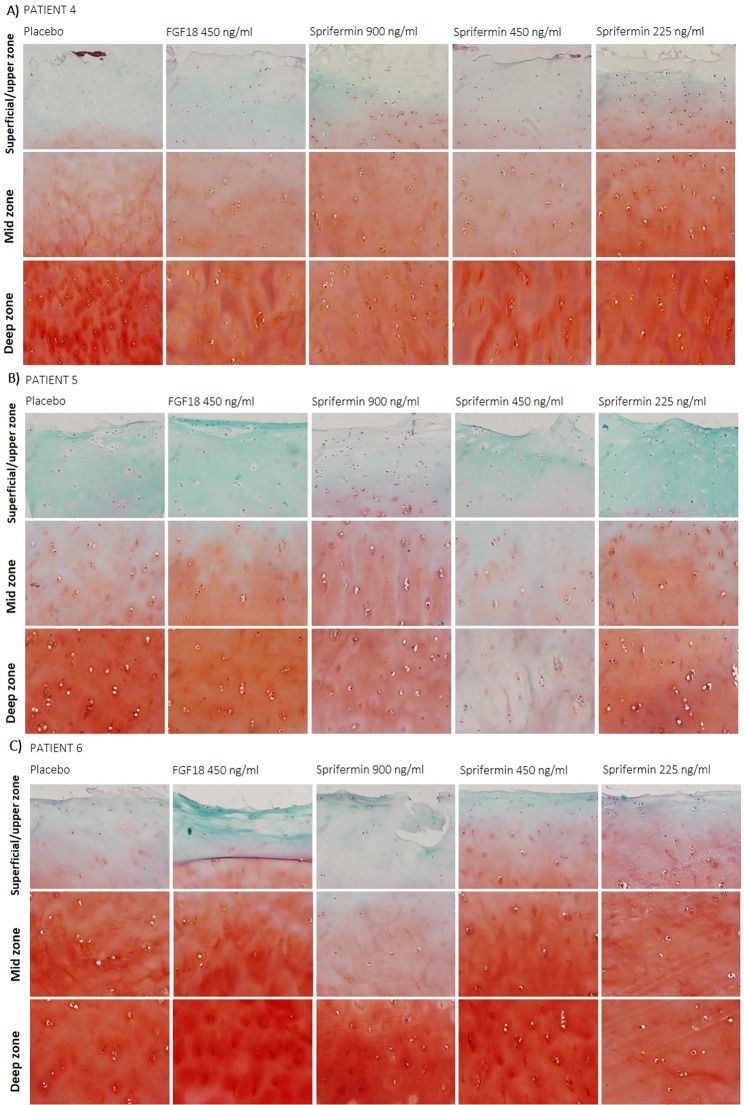
Proteoglycan (Safranin O/Fast Green) content at termination, individual patients.Proteoglycan content of human OA cartilage explants after 10 weeks of culture. Patient 4 (**A**), 5 (**B**) and 6 (**C**) were included. Proteoglycan content was evaluated by Safranin O/Fast Green staining of 5 μm sections of FFPE explants from termination of the experiment (day 70). Two replicate explants for each patient and treatment were stained, of which one representative is shown by pictures of superficial, middle and deep cartilage layer, respectively.

Cartilage explants from fixated and stained with fast green and safranin O at the day 70, to visually assess the proteoglycan content. Figure 3 depicts three representative patients and the superficial (upper), mid and deep zone (bordering to the calcified cartilage). Proteoglycan was gradually lost from the superficial zone to the deep zone, as observed by green to deep red color. In agreement with the gene expression analyses, no visual difference between treatment arms in red coloration of cartilage sections from different areas of the cartilage, could be observed at the end of the termination of the study (Fig. 3).

## Discussion

The data from this study suggests that sprifermin stimulates human articular cartilage from 11 knee OA patients undergoing total knee replacementin a temporal biphasic fashion that eventually results in production of hyaline cartilage. To summarize, the cartilage explants permanently stimulated with sprifermin had continuously increased metabolic activity throughout the culture period (day 0–70). Duringearly-phase culturing (day 7–28) the explants had increased aggrecanase-mediated aggrecandegradation (NITEGE) and decreased type II collagen formation (PIIBNP), whereas the late-phase showed increased type II collagen formation.This investigation builds on a previous study that investigate the effect on human and porcine monolayer chondrocyte cultures^11^ and bovine cartilage explants^12^. In current model we use OA human cartilage which is known to be more heterogenous than that of healthy bovine cartilage cultures. With current study, we aimed to support and confirm previous finding that there may be biphasic ECM remodeling effect of FGF18 and sprifermin on articular cartilage, using material that is translational to real life settings.

In the early phase of culturing, the chondrocytes of sprifermin-treated explants seemed to exhibit a catabolic phenotype, with decreased PIIBNP release and increased NITEGE release. The NITEGE release was previously seen in bovine articular cartilage, where it was suggested to be part of a coordinated process to expand the lacunae and allow proliferation of chondrocytes^12^. In the present study, it was not possible to monitor the temporal changes in cell proliferation. However, the increasing metabolic activity during the early phase could very well reflect a growing chondrocyte population. If the chondrocytes are actively proliferating in the early phase, then ECM formation should be compromised, assuming a negative linear correlation between proliferation and ECM formation as previously shown^11^. This would explain the decreased PIIBNP release. In previous published work it has been shown that proliferation is indeed induced by sprifermin; alginate cultures of human primary chondrocyte show up to a 4-fold increase in cell numbers when stimulated with sprifermin.

In the late phase of culturing, the chondrocytes of sprifermin-treated explants exhibited an anabolic phenotype. The aggrecanase activity responsible for the NITEGE release during early-phase culturing was gone, while type II collagen formation was increased. If ECM degradation is a prerequisite for chondrocyte proliferation, which in turn compromises parallel ECM formation, then the late phase seems to be the time where active proliferation has been completed and chondrocytes produce new ECM, which could also be reflected in the further increasing metabolic activity.

The sprifermin-induced ECM remodeling effects observed were superior to the effects of the natural ligand FGF18, potentially due to the roughly five times higher potency of sprifermin on the FGFR3 receptor. Meanwhile, the effect on metabolic activity of the explants were similar for sprifermin and FGF18 at the dose level tested in this study (450 ng/mL). Lower doses would maybe be able to differentiate the two.

To the best of our knowledge, sprifermin has proven to be anabolic in studies from bench to bedside. We therefore hypothesize that the catabolic effects suggested by the present study, are part of a coordinated sequential process leading to cartilage regeneration. Indeed, data obtained at termination of this study (day 70) show highly metabolically active chondrocytes (metabolic activity and expression of ECM molecules remains), with no indication of chondrocytes with an aberrant phenotype. The genetic markers could not discriminate between sprifermin and vehicle, potentially due to the indirect normalization to the number of cells (through the endogenous reference gene), in turn eliminating the effect of proliferation.

The biphasic process of ECM remodeling observed in this study may be indicative of the events occurring in the knees of OA patients treated with sprifermin in a clinical setting. Despite the limitations of the *ex vivo* model used in the present study, several translational parameters exist. (1) The articular cartilage specimens used in the present study were retrieved from knee OA patients scheduled for knee replacement surgery. Likewise, the OA patients enrolled in the latest clinical study have symptomatic radiographic knee OA, Kellgren-Lawrence grade 2 or 3. (2) The *ex vivo* model studies the articular cartilage, with chondrocytes residing in their natural matrix. (3) In both *ex vivo* and *in vivo* studies, sprifermin is added directly to the articular cartilage either by addition to the culture media or by intra-articular injections into the synovial cavity. (4) The dose levels used *ex vivo* are physiologically relevant.

If biphasic ECM remodeling occurs in the articular cartilage of sprifermin-treated knee OA patients, the NITEGE release might be interesting in several ways. First, one could speculate whether AGNx1 could be used as an early efficacy biomarker for sprifermin. In the present study, no clear correlation could be found between responders/non-responders of PIIBNP and early-phase NITEGE release. Data are very limited as only 2 patients (patient 6 and 8) did not respond by increased late phase PIIBNP. Second, the NITEGE fragment may have bioactive effects when released to the synovial cavity. This statement is based on the fact that a 32-mer fragment of aggrecan, containing the same C-terminal sequence (NITEGE373) as detected by the AGNx1 ELISA, has been shown to exert anti-anabolic, pro-catabolic and pro-inflammatory effects on chondrocytes, synovial fibroblasts and macrophages *in vitro*^23^. Such a potential bioactivity of a bi-product of sprifermin-induced cartilage regeneration, is obviously important for evaluation of effects and potential side effects observed in the clinic. However, the decreased release of the AGNx1 fragment at late-phase culturing might indicates that long-term effects may be limited.

The following limitations apply to the present study: Only articular cartilage was investigated without accounting for the influence of other joint tissues or mechanical stress. Moreover, the human OA articular cartilage had a high degree of variation, due to several factors. First, the cartilage was retrieved from OA patients, without selecting for disease-related factors, such as gender, age, obesity, inflammatory status etc. This introduces patient-to-patient variation. Second, the OA patients were end-stage with highly destructed cartilage, meaning that the isolated cartilage was taken from the limited areas of remaining cartilage. Therefore, the anatomic location and the thickness of the isolated cartilage varied. We have tried to accommodate the cartilage variation by including many patients in the study, and by revealing the individual patient data in the additional files (see Supplementary Figs. 1–4, Additional File 2). And lastly, temporal changes in cell proliferation could not be analyzed due to limited volume of cartilage from each patient. Instead, metabolic activity of the explants was monitored over time, to control for viability and to give an indication of potential cell proliferation in response to sprifermin.

## Conclusions

Sprifermin inducedbiphasic ECM remodeling in human knee OA articular cartilage *ex vivo*. This ECM remodeling was initiated by increased aggrecanase-activity and decreased type II collagen formation, followed by increased type II collagen formation. Meanwhile, the metabolic activity was continuously increased, potentially indicating a growing chondrocyte population in the early phase and increasing ECM production in the late phase.

## Declarations

### Ethics approval and consent to participate

The present study utilizes cartilage from patients with knee OA, obtained from the Department of Orthopedics at Gentofte University Hospital, Denmark. This was approved by the Danish National Committee on Biomedical Research Ethics, Department of Health, approval no. H-D-2007-0084. The retrieval procedure followed international ethical guidelines for handling human samples and patient information, and all participants signed informed consent prior to enrolment in the study.

## Supplementary information

Supplementary Dataset 1.

## Data Availability

All data generated or analyzed during this study are included in this published article and its supplementary information files.
