# Supplementary material for: Sprifermin (rhFGF18) versus vehicle induces a biphasic process of extracellular matrix remodeling in human knee OA articular cartilage ex vivo
Source: Sci Rep. 2020 Apr 7;10:6011. doi: 10.1038/s41598-020-63216-z (PMC7138815; doi:10.1038/s41598-020-63216-z)
Supplement: Supplementary file 1 — Supplementary Dataset 1. [file 41598_2020_63216_MOESM1_ESM.pdf]

Supplementary data for the manuscript entitled: Sprifermin (rhFGF18) versus vehicle induces a biphasic process of extracellular matrix remodeling in human knee OA articular cartilage *ex vivo*

D. Reker[1,2], A. S. Siebuhr[1], C. S. Thudium[1], T. Gantzel[3], C. Ladel[4], M. Michaelis[4], A. Aspberg[5], M. Berchtold[2], M. A. Karsdal[1], A. Gigout[4], and A. C. Bay-Jensen[1*]

SUPPLEMENTARY FIG. 1 METABOLIC ACTIVITY (ALAMARBLUE) OVER TIME, INDIVIDUAL PATIENTS.

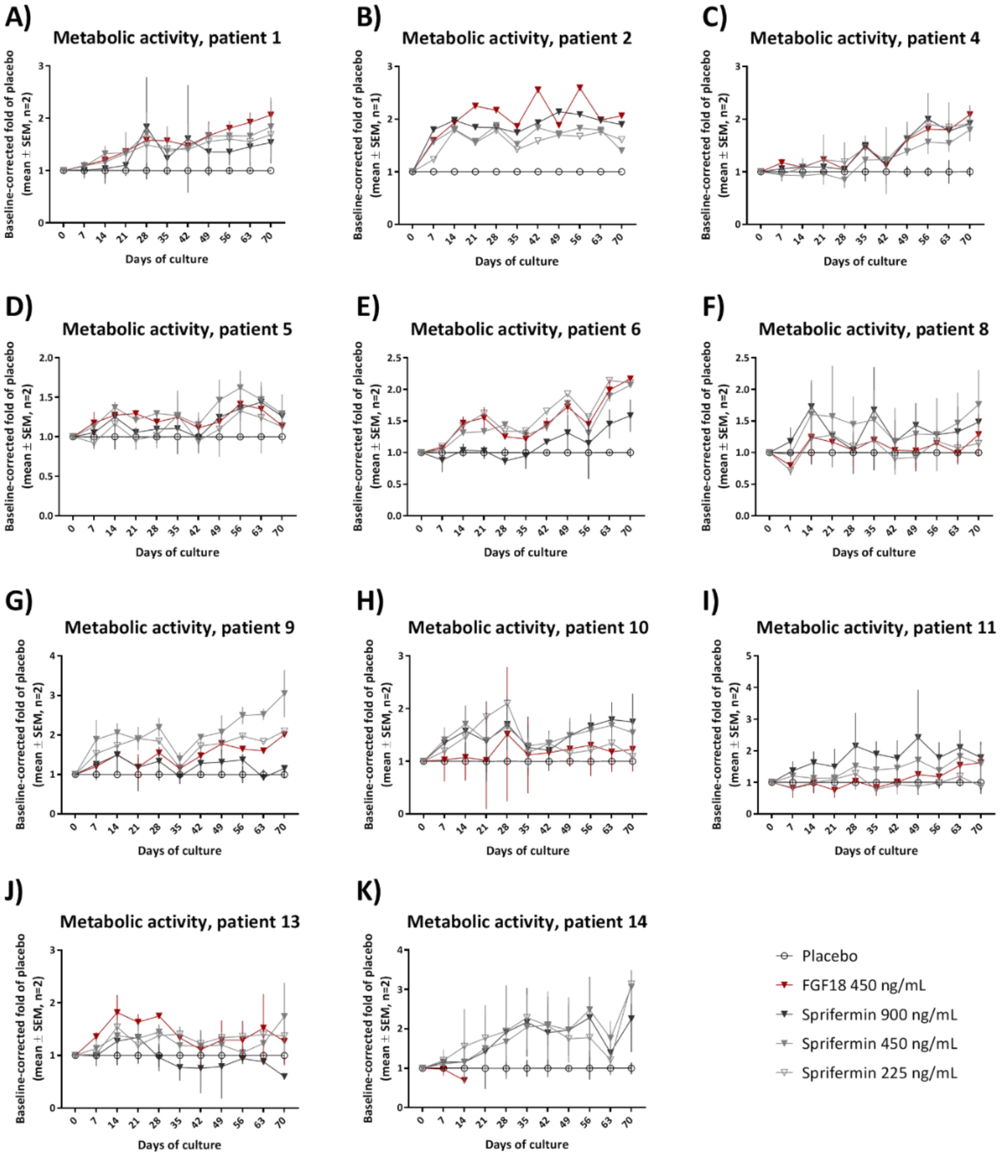

Metabolic activity of human OA cartilage explants during ten weeks of culture. Patient 1 (A), 2 (B), 4 (C), 5 (D), 6 (E), 8 (F), 9 (G), 10 (H), 11 (I), 13 (J) and 14 (K) are shown. Explants were incubated 3 h with 10% Alamar Blue®. Data was normalized to baseline (day 0) and presented as the fold from placebo. Values are mean ± SEM.

SUPPLEMENTARY FIG. 2 TYPE II COLLAGEN FORMATION (PROC2) OVER TIME, INDIVIDUAL PATIENTS.

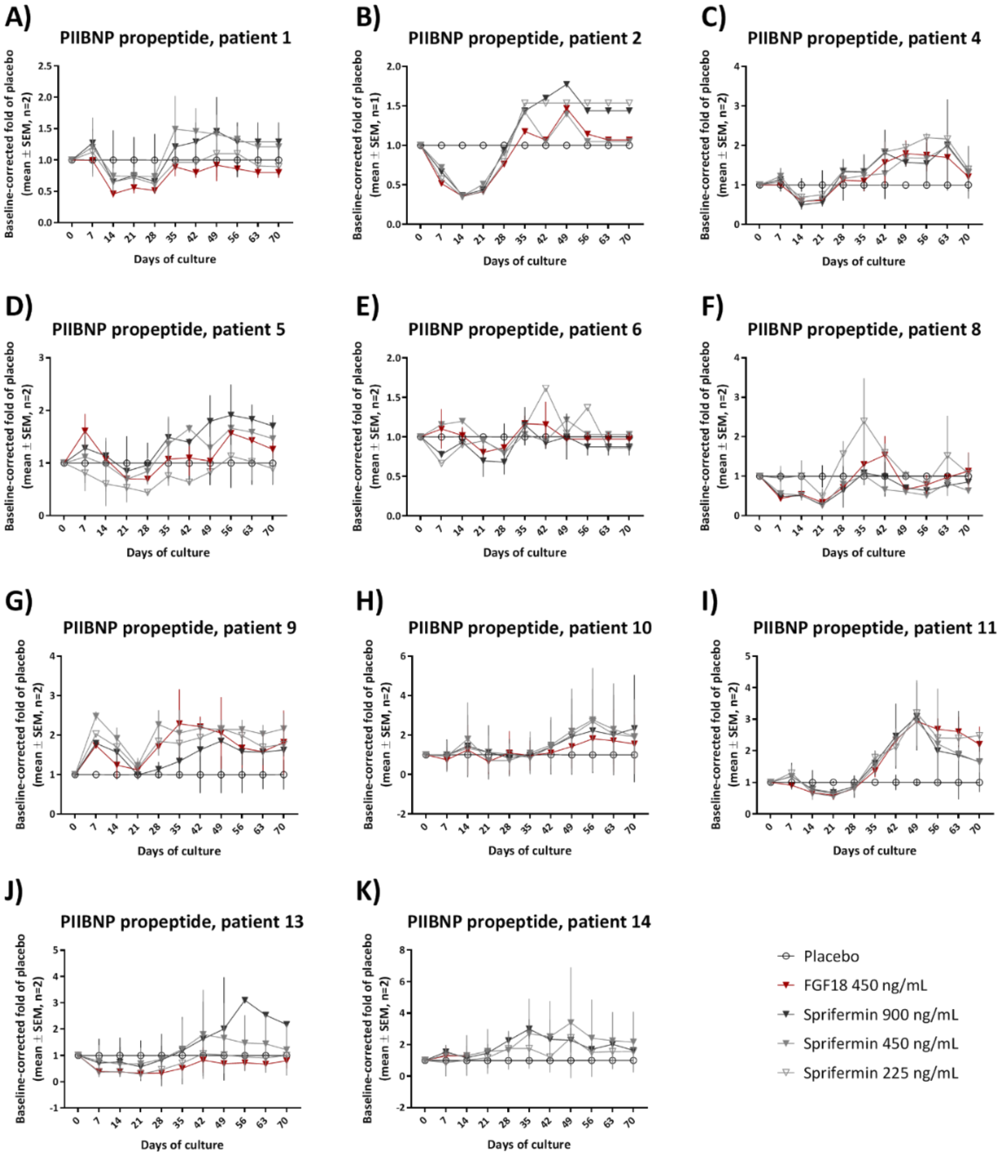

Type II collagen formation of human OA cartilage explants during ten weeks of culture. Patient 1 (A), 2 (B), 4 (C), 5 (D), 6 (E), 8 (F), 9 (G), 10 (H), 11 (I), 13 (J) and 14 (K) are shown. ProC2 was measured in conditioned media collected during culturing. Data was normalized to baseline (day 0) and presented as the fold from placebo. Values are mean ± SEM.

SUPPLEMENTARY FIG. 3 AGGRECANASE-MEDIATED AGGRECAN DEGRADATION (AGNX1) OVER TIME, INDIVIDUAL PATIENTS.

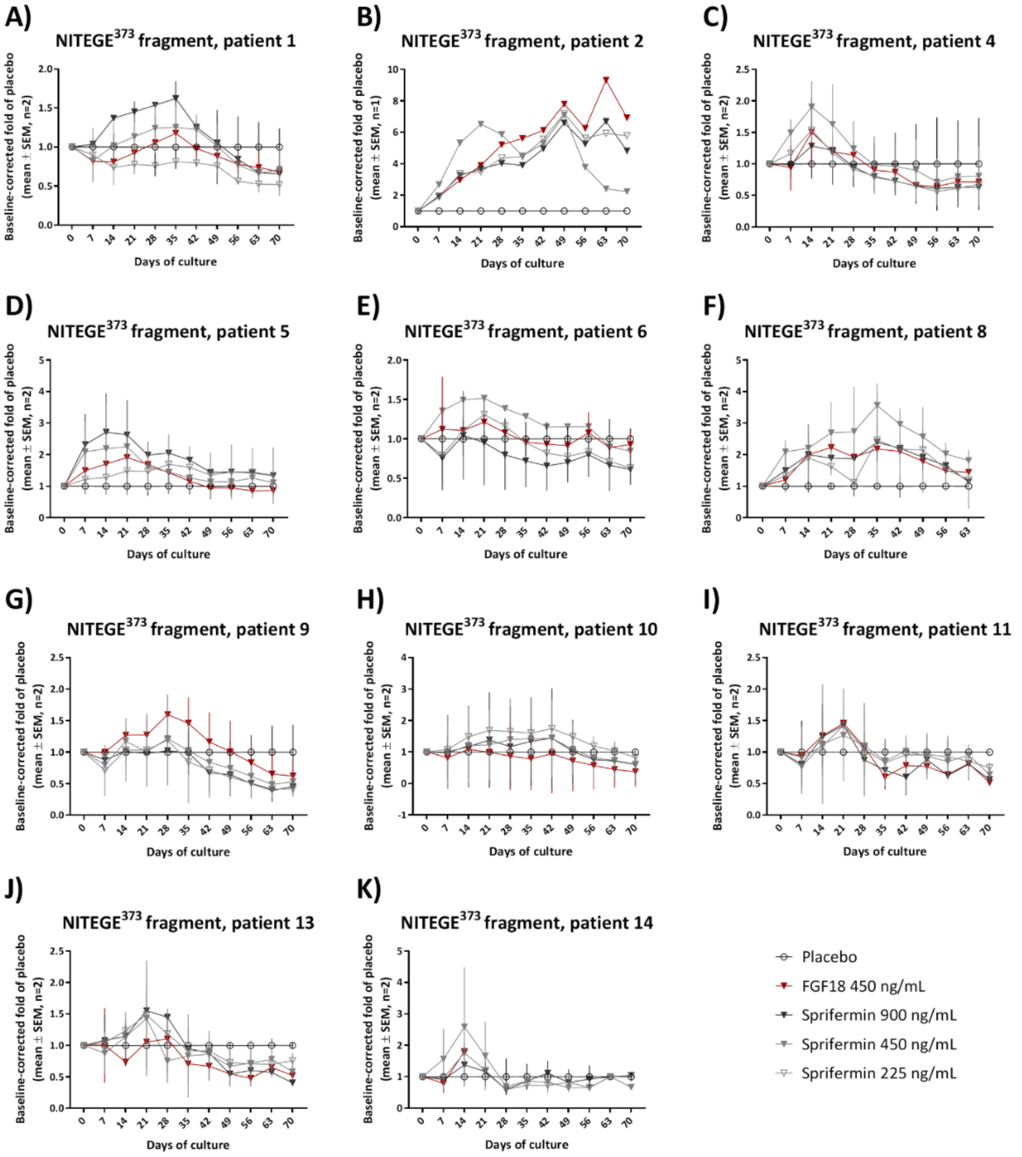

Aggrecanase-mediated aggrecan degradation of human OA cartilage explants during ten weeks of culture. Patient 1 (A), 2 (B), 4 (C), 5 (D), 6 (E), 8 (F), 9 (G), 10 (H), 11 (I), 13 (J) and 14 (K) are shown. AGNx1 was measured in conditioned media collected during culturing. Data was normalized to baseline (day 0) and presented as the fold from placebo. Values are mean ± SEM of two replicate explants.

SUPPLEMENTARY FIG. 4 GAG OVER TIME, PATIENTS COMBINED (*N* = 5).

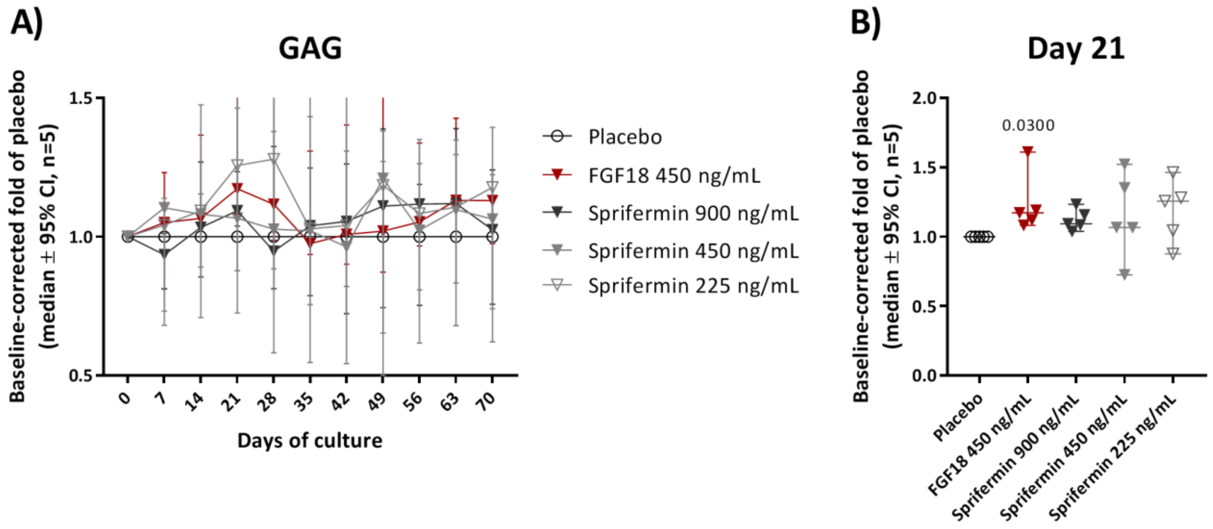

Glycosaminoglycan release from human OA cartilage explants during ten weeks of culture. Patient 1, 2, 4, 5 and 6 were included. GAG was measured in conditioned media collected during culturing. (A) The patient means (the mean of two replicate explants from each patient, calculated after baseline- and placebo-correction) were combined and presented as median ± 95% CI. (B) The medians ± 95% CI of day 21 are presented. Significance level to placebo was determined by Dunn's multiple comparisons test, and p-values ≤ 0.05 are indicated above each treatment group.

Glycosaminoglycan (GAG) assay. 1.9-dimethylmethylene blue (DMMB) dye solution was used for quantifying the total glycosaminoglycan (GAG) release1,2. Briefly the assay was prepared as following, samples were added alongside a known dilution of chondroitin sulphate A in a 96-well plate. The DMMB dye solution was added to all wells, and the colorimetric reaction was measured at 605 nm on a standard laboratory plate reader.

References

1. Farndale RW, Sayers CA, Barrett AJ. A direct spectrophotometric microassay for sulfated glycosaminoglycans in cartilage cultures. Connect Tissue Res 1982;9:247-8.

2. Ratcliffe A, Doherty M, Maini RN, Hardingham TE. Increased concentrations of proteoglycan components in the synovial fluids of patients with acute but not chronic joint disease. Ann Rheum Dis 1988;47:826-32.

Table S-1 SAMPLE OVERVIEW. ARTICULAR CARTILAGE FROM 14 OA PATIENTS WAS TESTED.

Patient no.	1	2	3	4	5	6	7	8	9	10	11	12	13	14
Placebo	2	1	1	2	2	2	2	2	2	2	2	2	2	2
IGF-I	2	1	1	2	2	2	2	2	2	2	2	2	2	2
FGF18 450 ng/mL	2	1	1	2	2	2	2	2	2	2	2	2	2	2
Sprifermin 225 ng/mL	2	1	1	2	2	1	2	2	2	2	2	2	2	2
Sprifermin 450 ng/mL	2	1	1	2	2	1	2	2	2	2	2	2	2	2
Sprifermin 900 ng/mL	2	1	1	2	2	2	2	2	2	2	2	2	2	2
Preservation	FFPE	FFPE	FFPE	FFPE	FFPE	FFPE	FFPE	FFPE	N_2	N_2	N_2	N_2	N_2	N_2

The table shows the no. of replicate wells (2 explants/well) included per patient and treatment. 11 patients were included in further analyses (highlighted in green). Patient 3 and 12 were excluded due to low IGF-I induction of ProC2 and patient 7 due to critical levels of metabolic activity from day 28. Patient 14 was included, but the FGF18 treatment group was excluded due to critical levels of metabolic activity from day 14. The sample preservation performed at termination of the study is indicated for each patient. FFPE, formalin-fixed paraffin-embedded; N2, Snap-frozen in liquid nitrogen and stored at -80°C.

SUPPLEMENTREY FIG. 6. VARIANCE IN METABOLIC ACTIVITY AT BASELINE IN THE 14 TESTED PATIENTS

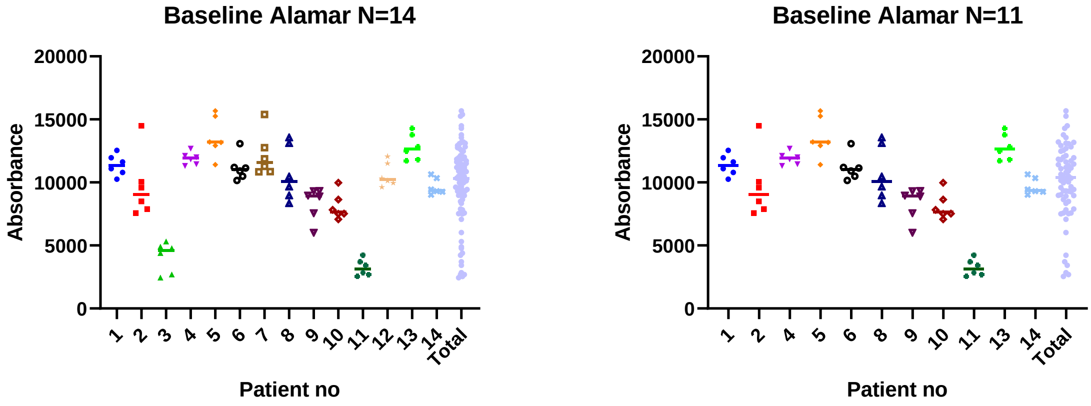

	1	2	4	5	6	8	9	10	11	13	14	Total
Number of values	6	6	6	6	6	6	6	6	6	6	6	66
Minimum	10253	7575	11323	11408	10159	8338	6021	7075	2537	11716	9018	2537
Maximum	12549	14490	12688	15673	13081	13555	9348	9968	4229	14284	10644	15673
Range	2296	6915	1365	4265	2922	5216	3327	2892	1692	2568	1626	13136
Mean	11378	9680	11907	13612	11161	10677	8360	8090	3237	12812	9667	10053
Std. Deviation	832.4	2543	492.7	1591	1020	2179	1317	1058	660.7	1043	660.1	3003
Std. Error of Mean	339.8	1038	201.2	649.3	416.3	889.4	537.8	431.9	269.7	425.7	269.5	369.6
